# Reliability and Reproducibility of Laser Speckle Contrast Imaging for Assessment of Superficial Perfusion in Patients with High-Risk Feet: A Feasibility Study

**DOI:** 10.3390/nursrep16080285

**Published:** 2026-08-15

**Authors:** Kathleen J. Finlayson, Emilia Maria Bran Hernandez, Emi Nishiyama, Jane O’Brien, Christina N. Parker

**Affiliations:** 1 School of Nursing, Centre for Healthcare Transformation, Queensland University of Technology, Victoria Park Rd, Brisbane, QLD 4059, Australia; christina.parker@qut.edu.au; 2 School of Clinical Sciences, Queensland University of Technology, Brisbane, QLD 4059, Australia; emilia.branhernandez@qut.edu.au; 3 Australasian College of Podiatric Surgeons (ACPS), Melbourne, VIC 3113, Australia; 4 St Andrews War Memorial Hospital, Brisbane, QLD 4000, Australia; 5 School of Health, University of the Sunshine Coast, Petrie, QLD 4502, Australia; jobrien5@usc.edu.au

**Keywords:** laser speckle contrast imaging, high-risk foot, superficial perfusion, reliability

## Abstract

**Background/Objectives:** Healthy macrovascular and microvascular circulation is essential for wound prevention and healing in high-risk feet. While validated methods of assessing lower limb macrocirculation exist, microcirculation assessment often relies on subjective clinical judgement. This study aimed to determine reliability and reproducibility of laser speckle contrast imaging (LSCI) of superficial circulation in the feet of adults attending a high-risk foot clinic. LSCI was undertaken on seven regions of the foot, and then repeated 3–5 min later. **Methods:** LSCI measures were compared to health and clinical measures using correlations, *t*-tests or ANOVA, as appropriate, and intra-class correlation coefficients (ICC) used to analyse reliability measures. In a sample of 31 participants, good test–retest reliability was found (3–5 min apart), with ICC from 0.94 (95% CI 0.76–0.98) to 0.99 (95% CI 0.96–0.99). **Results:** A sub-group of 10 participants were available to test reproducibility one week later, with Cronbach’s alpha ranging from 0.83 to 0.97. Lower LSCI perfusion measures were significantly related to longer ulcer duration, use of a walking aid, and presence of respiratory disease or previous cerebro-vascular accident. **Conclusions:** These findings support the potential role of LSCI as a non-invasive method to evaluate superficial perfusion in high-risk feet.

## 1. Introduction

The global burden of vascular disease, encompassing both macrovascular and microvascular pathologies, continues to increase and presents a significant public health challenge. According to the World Health Organization, cardiovascular diseases remain the leading cause of mortality worldwide, accounting for approximately 17.9 million deaths annually [1]. This growing burden is heightened by the rising prevalence of risk factors such as diabetes, hypertension, and ageing populations [2]. Among these, microvascular dysfunction presents unique challenges, possibly contributing to delayed wound healing, tissue breakdown, and chronic wounds that significantly impact patient quality of life and healthcare systems [3,4].

Effective circulation is essential for wound healing and prevention of tissue breakdown. Adequate microvascular circulation has been identified as related to progress in wound healing [5]. While assessment of macrovascular circulation has well-established methodologies, such as the ankle–brachial pressure index (ABPI) [6], the evaluation of microcirculation remains challenging. Current tools, including video microscopy, laser Doppler flowmetry, and transcutaneous oxygen measurements, provide insights into microvascular function, but are, however, rarely used in routine clinical practice due to their complexity, high costs, and limited accessibility [7]. As a result, clinicians often rely on subjective assessments of microcirculation, which are prone to variability and lack reproducibility.

Laser speckle contrast imaging (LSCI) has emerged as a useful non-invasive tool for objectively measuring superficial microvascular perfusion [8]. This technique utilises laser light projected onto tissue surfaces, creating a speckle pattern from interference [9]. Static tissues produce a stable pattern, while moving blood cells cause fluctuations that can be analysed to quantify perfusion [9]. LSCI provides real-time imaging of blood flow, making it particularly useful for assessing wound viability. It has shown success in acute settings, such as the early identification of poorly perfused areas in burn wounds that are visually undetectable at the time of imaging but eventually become non-viable [10]. LSCI’s rapid and objective assessment capabilities may facilitate early detection of perfusion deficits, reducing complications and improving outcomes for people with hard-to-heal wounds [11]. Studies of LSCI with patients with other conditions have reported that LSCI is highly accurate in monitoring perfusion [12], and reliable, with high convergent validity with thermography [13]. Although other imaging technologies have been developed, such as laser Doppler flowmetry, optical coherence tomography and portable optical diffuse speckle pulsatile flowmetry [14], LSCI requires a shorter time to obtain the images, is more user-friendly to put into practice and is focused on detecting blood flow [14,15], factors that make it more aligned with the needs of clinical wound assessment.

LSCI has emerged as a potential tool for managing chronic wounds, where its integration into clinical workflows could enable personalised treatment plans and improve resource allocation. Studies highlight LSCI’s ability to assess and quantify microvascular blood flow non-invasively, making it a valuable tool in visualising tissue perfusion. While research primarily focuses on peripheral arterial disease [16], the detailed perfusion data provided by LSCI suggests its potential to identify people at higher risk of complications, allowing clinicians to tailor interventions more effectively. Furthermore, the implementation of LSCI in wound care management may reduce healthcare costs, as after the initial investment in the equipment, it is inexpensive, and there appear to be no adverse effects [12].

This study aimed to investigate the feasibility, intra-rater reliability and reproducibility of LSCI for assessment of superficial perfusion of feet in community-living adults attending a high-risk foot clinic. By addressing the unmet need for an accessible and reliable microcirculation assessment tool, this research seeks to provide insights into the potential of LSCI to transform wound care management and improve outcomes in at-risk populations.

## 2. Methods

### 2.1. Research Design

An observational study of the reliability and reproducibility of LSCI measurements on the lower limbs of persons attending a high-risk foot clinic was undertaken.

### 2.2. Sample and Setting

All adults fitting the inclusion/exclusion criteria who attended a metropolitan outpatient high-risk foot clinic in Australia between August and November 2018 were invited to participate.

### 2.3. Participants

Inclusion Criteria: Adults attending a high-risk foot clinic, aged 18 years or older, with or without a foot ulcer, and sufficiently able to understand to give consent.

Exclusion Criteria: Persons with foot ulcers with malignancy present, and persons with cognitive impairment.

### 2.4. Data Collected

Data were collected from medical records on health and demographic characteristics, including age, sex, medical history, foot ulcer history, foot risk factors, foot ulcer characteristics (ulcer size, depth, duration, wound bed tissue type, and Texas wound classification), pedal pulses, toe pressures, lower limb tissue characteristics, peripheral neuropathy, and signs of infection. Laser speckle contrast imaging was performed utilising a high-resolution camera (PeriCam PSI System 2017), capturing up to 100 images per second. Outcomes were measured as arbitrary perfusion units as indicated by the LSCI software (PIMSoft 2017).

### 2.5. Process

A fixed focal length was used, with the camera on a small flexible arm. Protocols were developed to ensure consistency of measurements and included the environment, participant preparation and process for recording. The protocols specifically addressed the following:

Environment: Maintenance of constant room temperature (the outpatient high-risk foot clinic air conditioning ensured a consistent 23 ± 1 degrees Celsius temperature, which was checked and recorded at the start of each session); maintenance of constant, ambient lighting; minimal air movement (the same dedicated room was used for all testing, with no fans); use of the same equipment for each procedure (including bed and pillows); and calibration of the camera at the start of each day, with the same operator maintained to minimise inter-operator variance.

Participant preparation: For all testing, participants were given 15 min to rest and acclimatise to the room temperature before the imaging. During the required rest period, data was collected on name, age, sex, heart rate and blood pressure. Lower extremities were observed for any signs of circulatory impairment, and findings documented. A black mat was placed under the limb if the recorded area was greater than that of the skin to be assessed, to remove background noise, as per the LSCI camera manual. During recording, participants were asked to restrict their movement and refrain from speaking throughout the recording.

**Figure 1:**
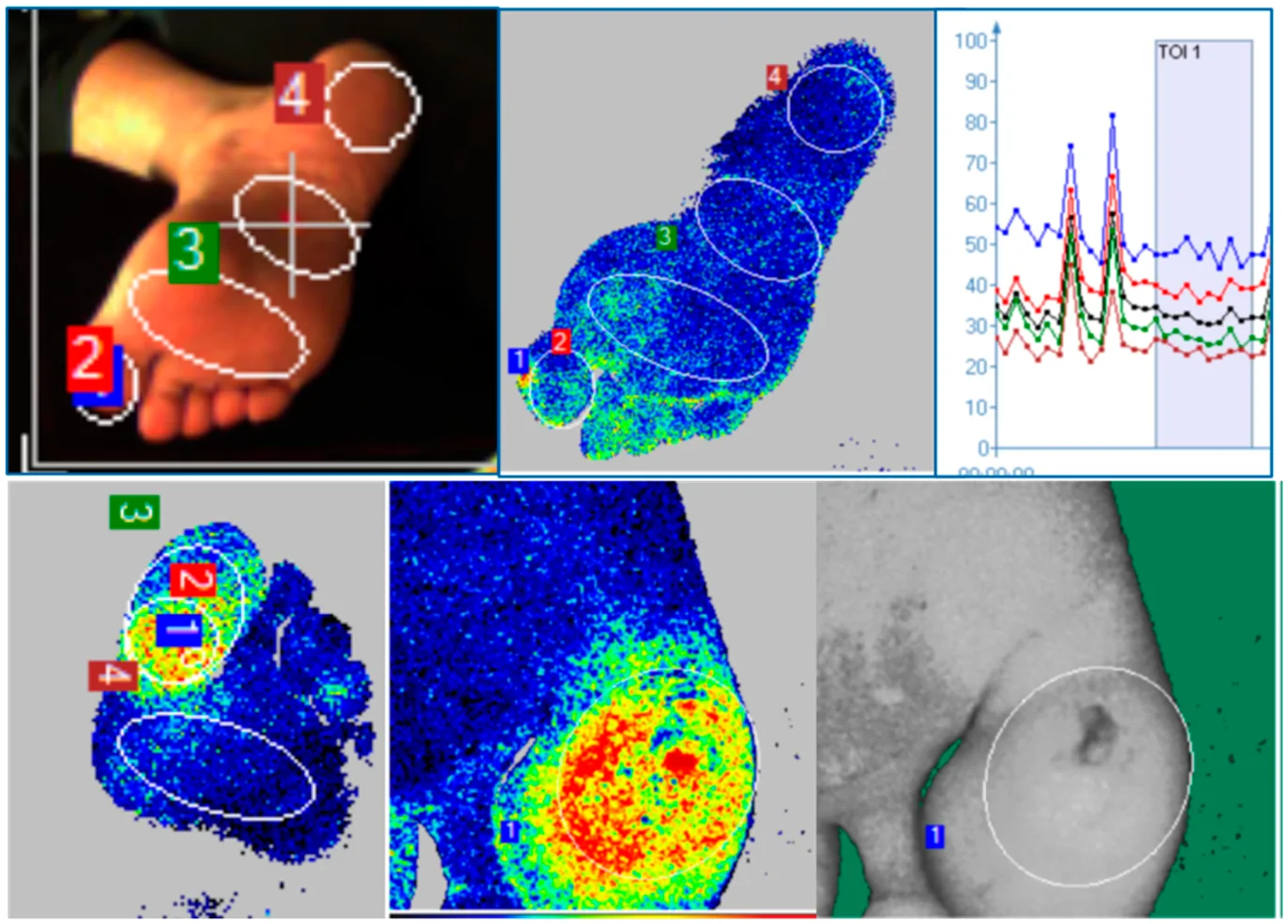
Examples of LSCI of plantar foot regions of interest (ROIs, including hallux, metatarsal heads, mid-foot plantar region and heel), clockwise from top left: plain photograph of foot with some ROIs identified and numbered; LSCI image of the whole foot, including the ROIs; software output of perfusion unit measures for each specific ROI (differing colours for each ROI), showing the time of interest used to determine mean measures, with X axis = time, and Y axis = perfusion units; plain photograph of the ulcer; LSCI of ulcer on plantar area of large toe; and LSCI of magnified foot ROIs.

Imaging process: The LSCI camera was positioned to have a set focal distance from the limb (30 cm) [17] and angled at 90° from the area, so that the camera head was parallel to the tissue being imaged. A study by Zotterman et al. [18] found no significant variance in perfusion observed for distances between 15 to 40 cm. The camera was set to take five images/second, taking an average of five images for each second to provide an effective frame rate of one image/second. The resolution was automatically determined when the distance was set. Image recordings of up to 60 s each were taken, or until waveforms stabilised for a minimum of five seconds (continuous), to minimise temporal variability (the time of interest), of the dorsal and plantar high- and low-pressure regions of the foot, including the hallux, meta-tarsal heads area, mid-plantar area, heel, mid-dorsum area, ulcer area and peri-wound area (for those with foot ulcers), see Figure 1. For test–retesting, the participant was then asked to move their position, the camera was re-set, and the participant position was re-set according to the protocol; another recording was then undertaken three to five minutes later. The process was repeated one week later to test reproducibility.

Data Analysis and Synthesis: The image recordings from the LSCI camera were analysed using the supplied software (PeriCam 2017). This software automatically calculated mean perfusion unit levels in the pre-specified regions of interest over specified periods of time and supplied the mean and standard deviations for each specified region/specified time. This data from the software was output in Excel format and then imported to SPSS v29 for further analysis. Descriptive statistical analyses were undertaken for all measurements of health and demographic characteristics. The LSCI measures were compared to all health-based and demographic characteristics, including clinical measures of peripheral vascular disease, ulcer severity, toe pressures and medical covariates, using correlations, *t*-tests or ANOVA tests, as appropriate. Minimal amounts of missing data were handled with listwise deletion. Intra-class correlation coefficients with 95% confidence intervals using two-way mixed effects models ICC (3,1), assessing consistency, were utilised to analyse test–retest reliability measures of the LSCI perfusion measurements.

### 2.6. Ethical Considerations

The study was performed in compliance with all laws and institutional guidelines and was approved by the Human Research Ethics Committee (HREC) of the university health centre involved, in accordance with the Code of Ethics of the World Medical Association (Declaration of Helsinki). Written informed consent was obtained from all participants.

## 3. Results

### 3.1. Sample Characteristics

A sample of 31 participants was recruited; the sample had a mean age of 66 years (SD 10.3). Around three-quarters (77%) were male. The most common health conditions were diabetes (97%), hypertension (81%), and cardiac disease (48%), and 19% required an aid for mobility. Fifty-two percent of the sample had an active open foot ulcer, which had been present for a median of 104 weeks (range 10–333 weeks). Around half of the sample had diminished or absent pedal pulses, and 77% (*n* = 24) of the sample had peripheral neuropathy. Further details on the sample’s characteristics are available in Table 1.

### 3.2. Feasibility

The feasibility outcomes of interest focused on the practicalities of undertaking LSCI within a busy clinical setting. In general, imaging of a particular area of interest took around five minutes, while for this study, completing the imaging of all areas of interest, followed by re-testing five minutes later, required around 20 to 30 min. The staff time required to set up the camera at the beginning of the day needed consideration, taking around 30 min. The study completion rate was 100%, as imaging took place on the same visit as obtaining consent. Participants were not asked to return to the clinic specifically for reproducibility testing, which was undertaken only if they were required clinically to return to the clinic in the following week.

### 3.3. Reliability/Repeatability of LSCI Perfusion Unit Measures

Repeatability testing, i.e., test–retest with the same tester and conditions, was undertaken as the form of reliability testing in this study. All participants had test–retesting undertaken (three to five minutes apart) for all regions of interest (ROI), as shown in Table 2 (e.g., see Figure 1). Intraclass correlations ranged from 0.94 (95% CI 0.76–0.98) to 0.99 (95% CI 0.96–0.99), demonstrating good test–retest reliability (see Table 2). A sub-group of participants (*n* = 10), who were required and able to return to the clinic, had repeat LSCI measurements taken on a return visit to the clinic around one week after the initial measurements. The characteristics of this sub-group had no significant differences relative to the larger group with regards to age, sex, mobility, comorbidities, medications, or medical history; however, they were more likely to have a foot ulcer present (90% (*n* = 9) of the sub-group, compared to 52% (*n* = 16) of the larger sample). Reliability testing found that Cronbach’s alpha ranged from 0.831 (95% CI 0.622–0.982) to 0.974 (95% CI 0.891–0.996) (lowest over the heel area).

### 3.4. Relationships Between LSCI Perfusion Units and Medical/Clinical Measures

The aim was to quantify relationships between LSCI perfusion units, clinical circulatory tests (i.e., pedal pulses, toe pressures), ulcer characteristics, and clinical and medical variables. Increased foot ulcer duration was found to be negatively correlated with LSCI perfusion units (rho −0.75, *p* = 0.002), while mean LSCI perfusion levels were lower in adults requiring a walking aid (*p* = 0.033, *d* = 0.86, 95%CI −0.06, 1.77); lower mean LSCI perfusion was associated with taking regular analgesia (*p* = 0.028, *d* = 0.86, 95%CI 0.09, 1.63), decreased LSCI perfusion levels were associated with concurrent respiratory disease (*p* = 0.049, *d* = 0.85, 95%CI −0.16, 1.85) and decreased mean ulcer LSCI perfusion was associated with history of a CVA (*p* = 0.01, d = 0.98, 95%CI −0.28, 2.21), see Table 3.

## 4. Discussion

The findings of this study highlight the potential role of LSCI in evaluating superficial perfusion in the assessment of foot health among people with high-risk feet. Assessment of the micro- and macrovascular status of a person with, or at risk of, a wound is critical, as most people with macrovascular disease develop concomitant microvascular dysfunction; so, although distinct, these are also complementary [19]. While macrovascular assessment has been well-understood and practiced, the microvascular assessment is less developed and utilised, despite its acknowledged importance [19].

To be clinically useful, any measurements should be reliable and sufficiently sensitive. The high intra-rater reliability of the LSCI measurements found in this study suggests that LSCI may be a valuable tool for assessing skin microcirculation, providing non-invasive perfusion data to help clinicians assess microcirculation more accurately than with observation. This study is limited by the lack of inter-rater reliability testing. Although there is evidence in the literature on the high inter-rater reliability of LSCI measurement in clinical settings [20,21], there is less reported on intra-rater test–retest reliability and reproducibility testing. Our results are supported by Mennes et al. [21], with a similar sample, who reported intra-observer agreement ICC of 0.711–0.950, compared to our 0.94–0.98. Schwartz et al. reported results of good reproducibility in testing using LSCI, with measurements taken between 48 h and one week apart, from healthy volunteers, in response to whole-body cooling [22].

The observed correlation in this study between prolonged ulcer duration and lower perfusion suggests the potential for early detection and intervention to mitigate the risk of chronic wound complications. The negative correlation indicates that LSCI could potentially detect changes in tissue perfusion before the ulcer progresses to be of long duration. These findings indicate the potential of LSCI as a reliable, non-invasive tool used to identify microvascular perfusion deficits in high-risk populations. Future prospective studies may identify its use in guiding personalised treatment strategies aimed at improving foot health and preventing DFU-related morbidity.

The use of walking aids was associated with lower LSCI perfusion levels at the metatarsal head, heel, and peri-wound areas, suggesting that reduced mobility and altered gait patterns may exacerbate perfusion deficits in critical weight-bearing regions [18]. This is clinically significant, as compromised microvascular perfusion in these areas increases the risk of diabetes-related foot ulcer development and impairs wound healing [4]. This highlights the importance of balancing load redistribution with the maintenance of adequate circulation to critical areas of the foot. The need for walking aids or loss of mobility may be used as an indicator of higher risk for compromised foot perfusion and subsequent foot ulcer development, necessitating targeted interventions like customised footwear, offloading devices, and/or proactive monitoring [23,24].

This study did not find a strong relationship between the LSCI units and pedal pulses or toe pressures. The inconsistency of pedal pulses as a measure of circulation has been well-reported [25]; thus, this finding is not surprising in this small sample. Mennes et al. [21] also reported the absence of significant relationships between LSCI and ankle–brachial index, toe pressures and TcpO2 measures, suggesting that the difference may relate to the difference between the arterial pressures and the microvascular flow. Further research is needed to investigate these relationships.

The primary limitation of this study is the small sample size, which was limited by the pragmatic aim to evaluate the feasibility of using repeated measurements from the LSCI camera in a busy clinical setting. The small sample size precluded multivariable regression analysis; thus, results must be considered with caution and as exploratory. Only a small sub-group of the sample had an appointment for the following week; in particular, this sub-group comprised those with open foot ulcers, thus limiting the size of the reproducibility testing. It follows that results regarding reproducibility must be considered with caution. Another limitation was that no correction for multiple tests was made, as this was an exploratory study, and all the individual *p* values are reported. This single-centre study was undertaken in a specialist high-risk foot clinic and its results should not be extrapolated to broader populations of individuals with diabetes. As an exploratory feasibility study, it was unable to evaluate the diagnostic accuracy of the LSCI measures against clinical outcomes.

## 5. Conclusions

Overall, the results provide valuable preliminary evidence on the reliability of LSCI in individuals with high-risk feet and suggest that LSCI has the potential for reliable clinical assessment of superficial perfusion of the foot. The dual assessment of microvascular and macrovascular health is particularly important in individuals at risk of or living with wounds, as impaired microcirculation can hinder wound healing, increase the risk of complications, and contribute to poorer clinical outcomes. Assessment of microvascular circulation through advanced imaging techniques like LSCI may support health professionals in decisions on timely interventions in the future.

## Figures and Tables

**Table 1:** Characteristics of the research sample.

Characteristic		Total Sample (*n* = 31) *n* (%)
Sex	Male	24 (77%)
	Female	7 (23%)
Age—years [mean (SD)]		66 (10.3)
Current smoker		2 (7%)
Requires walking aid		6 (19%)
Osteoarthritis		7 (23%)
Rheumatoid arthritis		3 (10%)
Hypertension		25 (81%)
Diabetes		30 (97%)
Cardiac disease		15 (48%)
History of CVA *		5 (16%)
Respiratory disease		5 (16%)
Renal disease		7 (23%)
Retinopathy		2 (6%)
Charcot foot		1 (3%)
Past surgery/trauma to study leg		5 (16%)
Insulin		14 (45%)
Antihypertensives		27 (87%)
Anticoagulants		17 (55%)
Regular analgesics		11 (36%)
Diminished or absent pedal pulses		16 (52%)
Peripheral neuropathy		24 (77%)
Foot ulcer present		16 (52%)
Site of foot ulcer: Hallux plantar		8 (53%)
Metatarsal plantar		5 (33%)
Toes		2 (13%)
Medial malleolus		1 (6.7%)
Clinical signs of ulcer infection (*n* = 16)		1 (6%)
		Median (range)
Ulcer duration (weeks)		104 (10–333)
		Mean (SD)
Toe pressure		97.6 (29.9)

* CVA: Cerebrovascular accident.

**Table 2:** LSCI perfusion unit repeatability measures, taken three to five minutes apart.

Region of Interest (ROI)	Mean (SD) LSCI Perfusion Units— 1st Measure	Mean (SD) LSCI Perfusion Units— 2nd Measure
Hallux *	68 (43)	70 (43)
Metatarsal head	56 (29)	55 (34)
Midfoot plantar	42 (15)	48 (15)
Heel	61 (40)	60 (32)
Dorsum	37 (13)	37 (10)
Ulcer (*n* = 16)	166 (108)	205 (145)
Periwound area	124 (36)	148 (38)

* *n* = 31, except for hallux (*n* = 24) due to amputations; and ulcer & periwound area only in participants with ulcers (*n* = 16).

**Table 3:** Relationships between LSCI perfusion units and clinical factors.

Clinical Measure		LSCI Measured Regions of Interest						
		Hallux	Meta-Tarsal Head	Mid-Foot Plantar	Heel	Dorsum	Foot Ulcer	Peri-Wound Area
Mean (SD) LSCI perfusion units		68 (43)	55 (29)	42 (15)	61 (40)	37 (13)	166 (108)	124 (36)
		Correlation values (* *p* < 0.05, ** *p* < 0.01)						
Toe pressure (mmHg)		−0.12	−0.11	−0.17	0.11	−0.42 *	0.16	−0.27
Foot ulcer duration		−0.75 **	−0.29	−0.20	−0.24	−0.64 *	−0.41	−0.21
		Mean (SD) LSCI perfusion units (* *p* < 0.05)						
Pedal pulses	Normal (*n* = 13)	73 (45)	70 (8.0)	50 (17)	62 (48)	46 (9.3)	117 (53)	117 (30)
	Diminished (*n* = 15)	67 (42)	57 (25)	43 (13)	64 (34)	40 (13)	178 (98)	123 (30)
	Absent (*n* = 2)	#	55 (35)	42 (17)	53 (45)	31 (12)	274 (232)	149 (77)
Uses walking aid	Yes (*n* = 6)	59 (56)	36 (13) *	37 (16)	36 (13) *	32 (16)	89 (25) *	36 (7.5)
	No (*n* = 25)	71 (39)	60 (30)	44 (15)	66 (42)	38 (12)	186 (113)	45 (13)
Cardiac disease	Yes (*n* = 15)	56 (27)	52 (26)	42 (14)	52 (30)	37 (13)	132 (58)	121 (35)
	No (*n* = 16)	78 (52)	59 (32)	42 (17)	70 (49)	36 (13)	218 (149)	128 (39)
History of CVA	Yes (*n* = 5)	65 (41)	54 (26)	41 (15)	60 (41)	37 (13)	80 (27) *	103 (36)
	No (*n* = 26)	82 (53)	64 (42)	47 (15)	65 (47)	38 (14)	188 (111)	130 (35)
Respiratory disease	Yes (*n* = 5)	63 (37)	67 (40)	42 (14)	58 (41)	28 (8.8) *	147 (81)	118 (30) *
	No (*n* = 26)	69 (45)	53 (27)	45 (21)	75 (40)	39 (13)	^	164 (57)

# There were no measures of the hallux area from participants with absent pedal pulses; ^ There was only one participant with a foot ulcer and without respiratory disease.

## Data Availability

Data can be obtained by reasonable request to the research team due to privacy or ethical restrictions.

## Public Involvement Statement

There was no public involvement in any aspect of this research.

## Guidelines and Standards Statement

This manuscript was drafted against the STROBE guidelines (www.strobe-statement.org) for observational research.

## Abbreviations

ABPI	Ankle–Brachial Pressure Index
CI	Confidence interval
CVA	Cerebro-vascular accident
ICC	Intra-class correlation coefficients
LSCI	Laser speckle contrast imaging
ROI	Region of interest
SD	Standard deviation
